# Definition of an Ontology Matching Algorithm for Context Integration in Smart Cities

**DOI:** 10.3390/s141223581

**Published:** 2014-12-08

**Authors:** Lorena Otero-Cerdeira, Francisco J. Rodríguez-Martínez, Alma Gómez-Rodríguez

**Affiliations:** LIA2 Group, Computer Science Department, University of Vigo, Galicia 32004, Spain; E-Mails: franjrm@uvigo.es (F.J.R.-M.); alma@uvigo.es (A.G.-R.)

**Keywords:** information fusion, ambient intelligence, context-awareness, smart city, ontology, ontology matching, multi-agent system

## Abstract

In this paper we describe a novel proposal in the field of smart cities: using an ontology matching algorithm to guarantee the automatic information exchange between the agents and the smart city. A smart city is composed by different types of agents that behave as producers and/or consumers of the information in the smart city. In our proposal, the data from the context is obtained by sensor and device agents while users interact with the smart city by means of user or system agents. The knowledge of each agent, as well as the smart city's knowledge, is semantically represented using different ontologies. To have an open city, that is fully accessible to any agent and therefore to provide enhanced services to the users, there is the need to ensure a seamless communication between agents and the city, regardless of their inner knowledge representations, *i.e.*, ontologies. To meet this goal we use ontology matching techniques, specifically we have defined a new ontology matching algorithm called *OntoPhil* to be deployed within a smart city, which has never been done before. *OntoPhil* was tested on the benchmarks provided by the well known evaluation initiative, *Ontology Alignment Evaluation Initiative*, and also compared to other matching algorithms, although these algorithms were not specifically designed for smart cities. Additionally, specific tests involving a smart city's ontology and different types of agents were conducted to validate the usefulness of *OntoPhil* in the smart city environment.

## 1. Introduction

The last decades have born witness to a sharp increase in the number of initiatives related to *Internet of Things and Smart Cities*. These initiatives are grounded in the idea that the Internet and e-related services can reach anywhere and therefore improve users’ lives with new types of services and comfort.

This sort of environment and its development is being supported not only within the research community but also at enterprise and governmental levels because of the repercussion it may have in the context of urban development policies. Thereby by integrating information and communications technologies (ICT) in the cities, two main purposes are addressed, to tackle problems related to common services, such as traffic or waste management [1], but also to provide the citizens with new services by adapting ICT to their needs.

With the aim of turning a city into a smart one, several steps must be taken [2]. First, the proper infrastructure must be deployed, including different types of sensors, smart devices and actuators, and also the networks that allow the communication among the different devices and systems. On top of this infrastructure different applications are placed. These applications are based on gathering data from the context and processing it, typically followed by some actuations on the environment according to the results of the processed data. Regarding this, the field of *Ambient Intelligence (AmI)* can be directly linked to smart cities, as AmI is devoted to proposing new and innovative ways of interaction between people and ICT [3].

One of the pillars where AmI is settled is precisely the use of context-aware technologies, where the context is considered as any information available to characterize the situation of an entity, given that an entity may be a person, place or object [4].

A smart city can be seen as an AmI-based distributed system where several agents on behalf of their users collect data from their contexts (integrated by users, other devices and the environment) by means of the different sensors. These data need to be properly fused and processed so that actionable information can be obtained from them, allowing an appropriate interaction of the users with the smart city. Given that multi-agent systems (MAS) are one of the most spread architectures for the development of distributed systems, they have been previously used in the context of AmI-based systems [5] achieving good results [3].

In smart cities this information about the context is provided by the different urban sensors and Sensor Networks (SNs) [1]. As these sensors are usually spread in a wide area and continuously measuring different variables, the amount of collected data is huge. All the sensed information of a smart city is usually obtained by means of different monitoring systems, and therefore a smart city can be thought of as a system of distributed systems [1] where public and private deployments coexist. It is also very likely that each one of these systems has its own software and hardware architectures, nevertheless to achieve a fully functional and efficient smart city it is mandatory that the information can be properly integrated and shared among the different systems [6].

This problem has been previously targeted and different alternatives have been studied, as in [6–8]. Some of the most commonly spread solutions do not approach this information integration problem directly but go around it by, for instance, forcing the use of a wrap for the different sensors or compelling the use of certain existing standards and protocols. Other efforts [9] make use of ontologies to semantically describe the available devices and services. The main drawback of this initiative is that authors miss to exploit the full potential of the ontologies and force a manual update of the ontologies’ descriptions whenever a new kind of device is added to the platform or when a new agent, not defined within the system, wants to take advantage of the city services or data.

Nowadays there are several spanish cities engaged [10] in the project of turning their cities into smart ones. One of such cities is A Coruña, whose initiative, *Smart Coruña* is an ongoing project currently being developed. This project is based on the platform *SOFIA*^2^ [11] that is being developed around *SOFIA (Smart Objects for Intelligent Applications)*, an european project to create a platform for *Internet* of *Things*.

The objective of the *SOFIA* platform is to allow the monitoring and control of a city environment, and actually improve the current monitoring and control capabilities of the city. This is done by exploiting the information provided by different sources in the city, such as legacy systems, sensor networks, and offer as well enhanced services to the citizens. This platform already comprises a large number of heterogeneous sensors that can be deployed statically or be embedded in mobile devices. However, in this platform the interoperability among the parties is guaranteed by limiting the definition of the ontologies of the different agents. Each agent can define its own ontology to represent the knowledge, however it must comply with one of the templates previously defined in the platform. The definition of the ontologies following the templates has to be done by the developer of the agent and previous to the registration of the agent in the system. From our point of view, this is the major drawback, as it turns the smart city into a closed system, since it is unable to interact with any agent that has an ontology that is not defined following the templates.

In this paper we propose an alternative approach that tackles the drawback previously mentioned and which is based on the use of *ontology matching techniques*.

The rest of the paper is organized as follows. Section 2, describes the use of ontologies in smart cities. Section 3 describes the requirements of ontology matching in smart cities. Section 4 introduces the related work. Then in Section 5 we describe our algorithm in detail, and in Sections 6 and 7 we provide, respectively, the evaluation and comparative results of the performance of our algorithm. Finally, Section 8 includes the main conclusions and remarks.

## 2. Using Ontologies in Smart Cities

**Figure 1.**
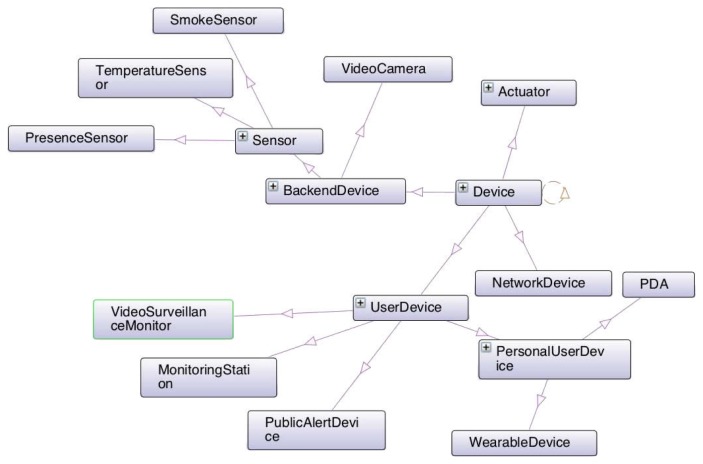
Snippet of the smart city's ontology.

The use of ontologies to describe smart cities’ knowledge is not new, there is for instance the case of the *SCRIBE* ontology *(Smarter Cities Reusable Information model and Business Events)* [12], which is a semantic model of a smart city, designed from the data gathered from several cities. However, as stated by its authors, this model is not closed and it may undergo several future changes that would need to be spread through the systems that may use it. Another example is *SOFIA* (Figure 1), as previously mentioned in Section 1, which is used to describe the available services and information in a smart city.

In this context of smart cities, the aim of using ontologies is twofold. Firstly, ontologies can facilitate the information fusion activity [13] of the data collected by the different agents so an integrated knowledge base for the smart city can be built. For this purpose, ontologies provide a way of understanding what is covered in the different information sources, *i.e.*, different sensors, and how their terms are used. This task of fusing the information compiled using different ontologies has been previously addressed in literature [14,15] and is proven to be successful approach.

Secondly, ontologies have proven their reliability in describing the meaning of concepts in a communication process [16] between the agents and therefore they are a way of reducing the semantic gap among the different parties. Guaranteeing this interoperability among the parties in such a system is a crucial point that needed to be addressed for a smart city project to succeed.

Nevertheless ontologies by themselves are not enough to guarantee the interoperability of the different parties, hence the approach we propose is to explore *ontology matching* techniques [17,18] as a possible solution for knowledge matching in smart cities.

Ontology matching helps in automatically generating new useful information from different information sources by integrating them. In our use case this feature has a direct use, for example, in service integration and discovery.

In a smart city there are agents that gather information from sensors, devices, other systems, *etc.*, and others that act on behalf of users to ask for services. Through these data gathered the agents get to know their contexts, so that they can adapt themselves to them and react to their changes. Additionally, agents can discover the services that the different systems in the smart city may offer. Regardless of the type of agent, if they are designed along with the ontology for the smart city, their inner-ontologies will already be compatible with that of the smart city. However, a smart city can, as well, have services provided by private enterprises and also agents that are integrated into the system in a later stage, or others that are itinerant and which only need a concrete service from the smart city at a certain time. In any of these scenarios, their information representations are not likely to be directly compatible with the one from the smart city and the firstly described agents. This causes the existence of a semantic gap between the knowledge representations of the different parties. To overcome this semantic gap we find the solution in the ontology matching techniques as they are a way of trimming the distance between the different knowledge representations by finding the correspondences between them. These techniques have been previously used in other fields such as biomedicine [19], geography [20] or P2P (Peer-to-Peer) networks [21], but to the best of our knowledge, their application in smart cities is a novel approach.

If an ontology evolves, the services built on top of it need to do accordingly to keep the model of the smart city open, therefore we point ontology matching techniques as the approach to follow to do this seamlessly. The alternative to this approach would be to manually reconcile the changes, *i.e.*, to have a human supervisor that would check the modifications in the ontologies, verify how these affect the existing services and consequently update the ontologies to reflect such changes. These changes range from an update in the name of a certain entity, to deeper modifications, such as hierarchical modifications in the structure of the ontology.

By using an ontology matching based approach we improve the scalability of the smart city since new agents, and with them new information sources and services, could be added transparently. These agent, even from the same type of previously existing ones, may have a different precision, scale, format, *etc.*, and therefore they can be considered as totally new ones.

In this section we have revised the use of ontologies in smart cities and laid the foundations of the use of ontology matching techniques. In the next section we further develop this approach and describe the requirements for ontology matching in smart cities.

## 3. Ontology Matching in Smart Cities

A smart city, as an open domain, is characterized by the existence of a large number of heterogeneous information sources where different services and agents must coexist and interact. From the ontology matching point of view the information in the smart city has two sides, a private and a public one.

The information in a smart city is described with an ontology, in our use case, *SOFIA*. Heretofore, the information in this ontology is publicly available for the different agents and deployments, however, it is expected that as the smart city grows, new specific subsystems are added. These subsystems may contain private information that they do not wish to share with other subsystems and agents. This limitation causes the existence of different layers of ontologies, which can be seen as different modules in the global smart city ontology. Regarding this aspect of the smart city, the matching process needs to be *modular*. An ontology matching algorithm in this context will deal with different types of situations: matching large ontologies to small ones, matching full ontologies with just snippets, *etc*.

The matching process also needs to be *lightweight*. Those agents that will seek and ultimately make use of the city's services may have been designed for other cities and therefore have different ontologies defining their structures. For these agents to seamlessly interact with the city's services, their ontologies need to be aligned but this process can not be heavy in terms of space and time consumption. As these agents are not native to the smart city but itinerant the results of their alignments will only be stored for as long as necessary. Additionally due to the nature of these agents’ requests, usually service demand, the answers must be shortly return, and therefore for these alignments the fact of retrieving a satisfactory short-term alignment is more important than achieving a long-term perfect one.

As for the matching process itself, it should avoid any kind of human input or supervision, as it would not be sustainable to manually reconcile and revise all the alignments produced. Regardless of the type of agent that interacts with the smart city, once the agent is deployed in the system it should be able to autonomously interact with it, obtaining or transmitting its essential information.

Considering the particular features of the ontologies used in this environment, it is worth noticing that the size of an ontology for a smart city is expected to be significantly bigger than the ontologies from the agents. Besides, these ontologies may not be balanced, *i.e.*, there could be a big difference in the amount of properties and classes that each one of them defines. In this sense, the algorithms that match such ontologies should be customizable to deal with these different situations.

In this section we have defined the requirements for a matching algorithm to be deployed in a smart city environment. Next section delves into the idea of a smart city as a distributed multi-agent system and outlines the matching strategies and the state-of-the-art matching systems.

## 4. Related Work

Ambient Intelligence encompasses nowadays a variety of computer science fields such as pervasive computing, artificial intelligence, sensor networks and multi-agent systems [5], as well as other fields outside the domain of computer science such as education or health, related to AmI due to the nature of existing AmI-based developments.

From the early definitions of AmI, *context-awareness, ubiquitous access* and *user interaction*, were identified as three of the main functionalities needed in an AmI-based system, although current trends require complementing them with others such as social intelligence [22]. One of the main problems when facing such a development is obtaining an integrated and coherent knowledge of the context that could be transparently used by different parties. This is so, because every sensing device perceives different stimuli and may process them in different ways. This is also one of the main concerns when designing a smart city. Such task must also be approached considering always that these are open systems modification and expansion prone. Therefore it is mandatory to provide the AmI-based systems with tools that allow the future modifications seamlessly.

As stated in [7], one of the worst problems of such these systems is the creation of *islands of services*, *i.e.*, having different services of different providers available to the users but so lacking of cohesion that would unnecessarily hinder the user's relation with the system. Additionally an agent moving from a smart city into a new one would be unable to function as the communication protocol and information representations would be unknown.

In order to avoid these and similar situations, in this paper we make use of ontology matching techniques as a way of allowing the fusion of the existing data gathered by the different sensors, obtaining this way a fully interoperable system in a transparent manner for the user.

This proposal lies its foundations on some previous works relating the fields of AmI and MAS such as [3,23], and expand them by integrating the ontology matching techniques as a way to perform information fusion among the different information sources. Multi-agent paradigm conforms to the AmI requirements as MAS can be seen a metaphorical representation of a human environment. Besides MAS offer further advantages such as *decentralized control* and *support of complex interactions between agents* by means of semantic specifications [24], useful in our case to accomplish the information fusion task.

When addressing the problem of information fusion, namely accessing and integrating the information sources, the main drawback is to understand what is covered in the sources and how the terms are used [25]. Regarding this, ontologies offer several advantages when used for information fusion tasks since they provide with a vocabulary describing a domain and a specification of the meaning of the terms in that vocabulary [17]. Besides the language used to specify the ontologies is expressive and allows querying.

As we stated before, the main purpose of ontologies in information fusion is to explicitly describe the content of the information sources. However, ontologies may be exploited in different ways. In [26], Wache *et al.*, define three different types of approaches for exploiting ontologies: *single, multiple* and *hybrid*, which respectively define one shared ontology for all the parties involved, one different ontology for each involved party, or a global shared ontology as well as different individual ones.

In an environment that follows a *multiple ontologies approach*, if the common mapping (alignment) between the ontologies of the different parties is not provided by external resources, there is the need to compute it by running an *ontology matching* tool.

There are different methods to compute the alignment between two ontologies. Such methods are habitually classified as [18] *terminological*, those based on string comparison [27], *structural*, those based on exploiting both the internal and external structure of the entities found in the ontology, *extensional*, those devoted to analyzing the set of instances of the classes (extension), and *combinational*, those that combine several of the other methods.

This classification is made according to the *kind of input* which is used by the elementary matching techniques [28]. Nevertheless, there are other classifications which consider different matching dimensions.

As seen in [17], another classification can also be made according to the *granularity* of the matcher and then to the interpretation of the *input information*.

According to *granularity*, matchers can be classified as *structure-level matchers* and *element-level matchers* which respectively compute the correspondences by analyzing how entities display in the structure of the ontology or by just considering the entities without their relations with others.

Additionally, according to the *input interpretation* matchers can be classified as *syntactic*, those that limit their input interpretation to the instructions stated in their corresponding algorithms, *external*, which exploit resources such as thesaurus or human knowledge to interpret the input or *semantic*, which use some formal semantics to interpret their input.

These classifications for ontology matching techniques are mostly derived from classifications previously made for schema matching approaches such as those by Rahm and Bernstein [29] or Do, Melnik and Rahm [30], and from studies in the database field [31,32]. In fact, the ontology matching field has its origins in the database field from which it inherits several concepts and techniques, although it has evolved independently.

This independent evolution is reflected by the amount of surveys and books that specifically reviewed the ontology matching field in the last decades such as [28,29,33–38], and also, by the number and variety of systems that address the ontology matching problem.

According to the classifications previously introduced, *OntoPhil* can be defined as a *combinational* approach to ontology matching since it includes both *terminological* and *structural* methods. Regarding the granularity of the matchers, there are *element-level* and *structure-level* matchers implemented within *OntoPhil*. At the same time, focusing on the input interpretation, there are *syntactic* and *external* matchers.

Some of the systems that have appeared in these years have participated in the Ontology Alignment Evaluation Initiative (OAEI) campaigns [39] which is an initiative that aims at evaluating the ontology matching tools. As for the field we are developing our algorithm, smart cities, it does not exist a proper benchmark test, we have turned to OAEI as it is recognized in the academic field as a prominent initiative in evaluating ontology matching systems and algorithms. Nevertheless, we have designed an experiment to test *OntoPhil with the SOFIA* ontology and 39 ontologies defined for different types of external agents, which is further detailed in Section 6.2.

### 4.1. Revision of Existing Systems

In its latest edition, *OAEI* 2013, 23 participants evaluated their algorithms in the different offered tracks. Some of these systems are outlined next.

AML (AgreementMarkerLight) [40] is an ontology matching framework based on element-level techniques and external resources as background knowledge. CIDER-CL (Cross-lingual CIDER) [41] is the evolution of CIDER (Context and Inference baseD ontology alignER) [42] which has been redesigned to include more features in the comparisons and new metrics for similarity computation such as a specific cross-lingual metric. CroMatcher [43] is an ontology matching system that includes different basic matchers both terminological and structural whose results are aggregated to produce the alignment of the input ontologies. Hertuda [44] is an element matcher that uses the Damerau-Levenshtein distance [45,46] for string comparison. HotMatch (Hands-On Training Match) [47] implements several basic matching strategies both element-based and structure-based. A set of filters are used to remove the incorrect correspondences and compose the final output. IAMA (Institute of Automation's MAtcher) [48] is a matching system specifically designed to deal with large ontologies by means of several similarity measures as well as filtering techniques. Lily [49,50] combines different matching strategies to adapt itself to the problem being tackled at each moment, generic ontology matching (GOM) for the normal-sized ontologies and large scale ontology matching (LOM) for more demanding matching tasks. LogMap (Logic-based and Scalable Ontology Matching) [51,52] initially computes a set of correspondences known as anchor mappings which are almost exact lexical correspondences with a confidence value assigned. Next, mapping repair and mapping discovery steps are iteratively applied to refine and discover new mappings. LogMapLt (LogMap Light) [53,54] is a lightweight version of LogMap, which skips some of the steps that LogMap defines. MaasMatch [55,56] initially used a combination of a string similarity measure and their own WordNet similarity to provide the matching between the ontologies. However, MaasMatch has lately included more complex string measures as well as structural similarities to its matching process. MapSSS [57,58] sequentially applies syntactic and structural metrics once the ontologies are represented as graphs. MapSSS designers aimed at adding also a semantic metric to improve algorithms performance. ODGOMS (Open Data Group Ontology Matching System) [59] combines element-level and structural-level techniques reinforced by optimization strategies. OntoK (Ontology Kernel) [60] uses both lexical and structural similarities. To compute the structural similarity it defines a entity graph that represents all the relations that an entity has with other entities. RiMOM2013 [61] is an evolution of RiMOM (Risk Minimization based Ontology Mapping) [62] that integrates different matching strategies which are automatically selected and combined in order to achieve the combination that better fits each matching problem. ServOMap [19,63,64], as first step, dynamically generates a description of each entity in the ontologies, which is used to compute the lexical similarity among the entities as another entry of the vector of terms that represents each entity. Next, a context-based similarity value is calculated by computing the similarity of the surrounding concepts for each entity. SLINT++ (Schema-independent Linked Data Interlinking System) [65,66] is a data interlinking system specifically designed to perform instance matching. It uses statistical measures to identify predicate alignments among the data sources. SPHeRe (System for Parallel Heterogeneity Resolution) [67,68] offers a different approach to ontology matching, as it exploits parallelism over multicore cloud platforms to perform the matching. StringsAuto [58] it is a syntactic matcher embedded in MapSSS, based exclusively on the use of string distance measures. Synthesis (Synthesizing Ontology Alignment Methods Using the Max-Sum Algorithm) [69] is a matching platform that combines different matching methods under a model-based synthesis framework. WeSeE-Match (Web Search Engine based Matching) [70,71] is an external approach which uses a web search engine for retrieving the relevant web documents for the concepts in the ontologies matched. WikiMatch [72] is also an external method which uses Wikipedia's [73] search engine to retrieve the relevant articles describing the concepts in the ontologies matched. XMapGen (eXtensible Mapping using Genetic) [74] and XMapSig (eXtensible Mapping using Sigmoid) [74] are variants of XMAP++ (eXtensible Mapping) [75]. These implementations include several matchers that compute the similarities between the terms from the input ontologies. The matchers used are linguistic-based, structure-based and they also exploit external resources. XMapGen uses a genetic algorithm to aggregate the different measures while XMapSig uses a sigmoid function. YAM++ ((not) Yet Another Matcher for Ontology Matching Task) [76–78] implements several matching approaches inherited from machine learning and graph matching. In a later phase, information retrieval approaches were also considered.

These systems are state-of-the-art examples of different strategies to ontology matching, however, none of them was specifically oriented to be deployed in a environment as a smart city.

Just considering the algorithms that took part in the OAEI-13, the one that shares more similarities with ours is *LogMap*, although it is not the only one in literature that follows the same general outline. Other examples are: *Anchor-Flood* [79], *Anchor-Prompt* [80], *ASCO* [81], *Eff2Match* (Effective and Efficient ontology matching) [82] and *SoBOM* (Sub-Ontology based Ontology Matching) [83]. In all these systems, first some initial alignments are computed which are then used as a basis to continue obtaining new alignments between the input ontologies. Actually, the approach that we describe in this work is motivated by some of the ideas studied in these approaches, although there are fundamental differences in the way some structures are exploited and considered. A brief review of these algorithms is included next.

*Anchor-Flood* starts off an anchor that is provided by a program module which is not considered part of the basic algorithm. This module uses both lexical and statistical relational information to obtain some aligned pairs. Then, taking advantage of the locality principle in the ontology graph, it analyzes the neighboring information of these aligned pairs (anchors) obtaining for each one a pair of blocks that contain possible similar concepts between the ontologies. These concepts are then aligned using lexical, semantic and structural information to discover new pairs, which are later further processed.

*Anchor-Prompt* takes as input a group of related terms (anchors), that can be both user-defined or automatically discovered by a lexical matcher. These pairs are considered as nodes in a sub-graph of the ontology. Anchor-Prompt analyzes which classes appear with a higher frequency in the paths that connect these related terms, as they are considered as potentially new similar concepts. The similarity measure of the detected pairs it founds in the different paths comes from an aggregation of all the measures from all the paths where these can be found. It also uses a filter to dismiss those pairs whose similarity measure does not reach the median of the similarity of all the identified pairs.

*ASCO* computes the alignment by applying TF/IDF (Term Frequency/Inverse Document Frequency) techniques and relying in WordNet as external resource. It is divided into two phases, a linguistic and a structural one. In the linguistic phase three different values are obtained by calculating the similarity values of names, labels and descriptions, which are then combined to provide the linguistic similarity of the input entities. Each one of these values is computed in a different way, while for names and labels, string metrics and WordNet are used, for the similarity of the description, TF/IDF techniques are used. For the structural phase, the authors follow the idea that if the paths from the root of two classes (*C_O_*_1_ and *C_O_*_2_) in the two hierarchies contain similar concepts, then *C_O_*_1_ and *C_O_*_2_ are likely to be similar too. To output the final alignment it combines the structural and the linguistic values and it returns those that exceed a threshold.

*Eff2Match* identifies the anchors by using an exact string matcher that considers both the names and labels of the entities. Next, using a VSM (Vector Space Model) approach, it lists candidates for those entities of the source ontology that have not been matched in the initial step. Namely, for each class, it obtains three vectors from the names, labels and comments (annotations) in the ancestors, descendants and the concept itself. Regarding properties, the vectors include the annotations for the property's domain and range classes as well as its own annotations. The similarity between two concepts is then calculated as an aggregation of the cosine similarity between the classes, ancestors and descendants vectors. In a subsequent phase of anchor expansion, new pairs are identified by applying terminological methods to compare source and candidate entities.

*LogMap* initially computes a set of correspondences known as anchor mappings which are almost exact lexical correspondences with a confidence value assigned. Next, mapping repair and mapping discovery steps are iteratively applied to refine and discover new mappings. To obtain the start anchors the algorithm previously computes a lexical and a structural index of the ontologies, which are also used to assign the confidence value of each anchor. From the initial anchors, new ones are discovered by using the ontologies’ extended class hierarchy.

*SOBOM* starts off a set of linguistically matched anchors. To obtain these anchors the textual information (names, labels, *etc.*), the structural information (number of antecesors, descendants and constraints) and the individual information (number of existing individuals) is taking into account. Out of these anchors the algorithm obtains sub-ontologies and ranks them according to their depths. To obtain the concept alignments, it computes the similarity between the different sub-ontologies obtained from the input ontologies according to their depths. Finally, it uses the previously obtained concept alignments to retrieve the relationship alignments.

In this section we have presented and defined the context for our algorithm, a smart city. We have also briefly presented some classifications for ontology matching approaches according to the literature, and situated our algorithm within these classifications. Then, we have revised some of the algorithms implemented or improved in the latest years, and as none was found that was devoted to the smart city field, we have further studied those that shared a similar outline with ours. In the following section, further details of our algorithm are provided while the comparison of our algorithm with those similar ones is included in Section 7.

## 5. An Ontology Matching Algorithm in AmI Based-Systems

In this section our ontology matching algorithm for smart cities is described. This algorithm proposes a method for automatically transforming the information from different source ontologies into a single representation, an alignment. The algorithm was defined to compile all the requirements necessary to be useful for the reference scenario described in Section 1. In this scenario the different agents in the smart city interact with it, either obtaining information from it, or transmitting to it the data gathered from the context. It may occur that an agent not defined for this smart city wants to use some of the knowledge in the smart city, access some services or share some information. To ensure that this will be possible every agent is provided with an ontology and the main terms in the smart city are as well included in another general ontology. When an external agent arrives into the smart city, it will only need to match its inner knowledge (semantically described in its ontology) with the city's ontology, in case it wants to access to some information or with another agent's ontology in case the latest offers a service the first wants to use. The algorithm described in this work is devoted to identifying the correspondences between such ontologies.

In this section we initially describe the integration of *OntoPhil* within a smart city. Then, we detail the different steps and modules in our algorithm.

### 5.1. Integration of OntoPhil in a Smart City

The *SOFIA*^2^ platform for smart cities lays the foundations of the interoperability among the different parties, known here as *KPs* (*Knowledge Processors*), in the ontological definition of the information that they exchange. These KPs could be any user, device, application or system that produces or consumes information from the platform.

The approach chosen in this platform to guarantee the seamless information exchange among the parties is to previously restrict the ontologies that the KPs use. Each different KP can define an ontology to represent its knowledge, however it must comply with one of the templates defined in the platform. From our point of view, this is the major drawback of this platform, as it limits the expressiveness of its KP and it is closed, since it is unable to interact with any KP that has an ontology that is not defined following the templates.

To overcome this situation and guarantee that other (external) KPs could also interact and take advantage of this platform, we have defined the algorithm presented in this work. To validate the assumption that it could be integrated in a smart city as *Smart Coruña*, we have defined and conducted a series of tests which involve, on the one hand the *SOFIA* ontology, and on the other hand different types of KPs. Such evaluation tests are further detailed in Section 6.2.

The integration of *OntoPhil* in a smart city is summarized in the following steps.

(Step 1) Let us consider that a new sensor agent, *Agent#A*, needs to transmit a measurement to the smart city (For the purpose of this section, we will consider just agents designed outside the smart city). The knowledge of this agent is represented by means of the *Ontology_Agent#A*. This ontology must be aligned with the ontology from the smart city *Ontology_SC* since this is the first time that *Ontology_Agent#A* interacts with the smart city.
(Step 2) To align these ontologies, an *Agent#OntoPhil* is triggered, which integrates the ontology from the smart city. This agent is responsible for running the matching algorithm between *Ontology_Agent#A* and *Ontology_SC*.
(Step 3) As result of running *OntoPhil*, an alignment between both ontologies is obtained, *Alignment* (*Ontology_Agent#A,Ontology_SC*). This alignment identifies those entities in both ontologies that are semantically equivalent. With this set of correspondences, the measurement in *Agent#OntoPhil* can be read and integrated in the smart city.
(Step 4) This alignment is stored in the smart city to be used for future interactions with *Agent#A*.
(Step 5) The next time that this *Agent#A* needs to transmit or obtain information from the smart city. The matching process is avoided as the corresponding alignment is already stored in the smart city.

### 5.2. Outline of the Proposed Algorithm

This algorithm lays its foundations on the exploitation of a set of some initial correspondences or *binding points* that connect one ontology to the other.

We aim at discovering a set of *binding points* between the input ontologies by using some new lexical matchers. Thereafter, taking these *binding points* as pivots, particular features and properties of the ontologies are exploited to discover new *binding points*. When these *binding points* are selected and refined, we obtain the alignment between the input ontologies which integrates the knowledge from both source ontologies.

The process designed takes a couple of ontologies as input and it applies, first some lexical matchers and later some structural matchers to obtain the final result. Therefore the followed matching process is *sequential* as defined by Euzenat and Shvaiko in [17].

**Figure 2.**
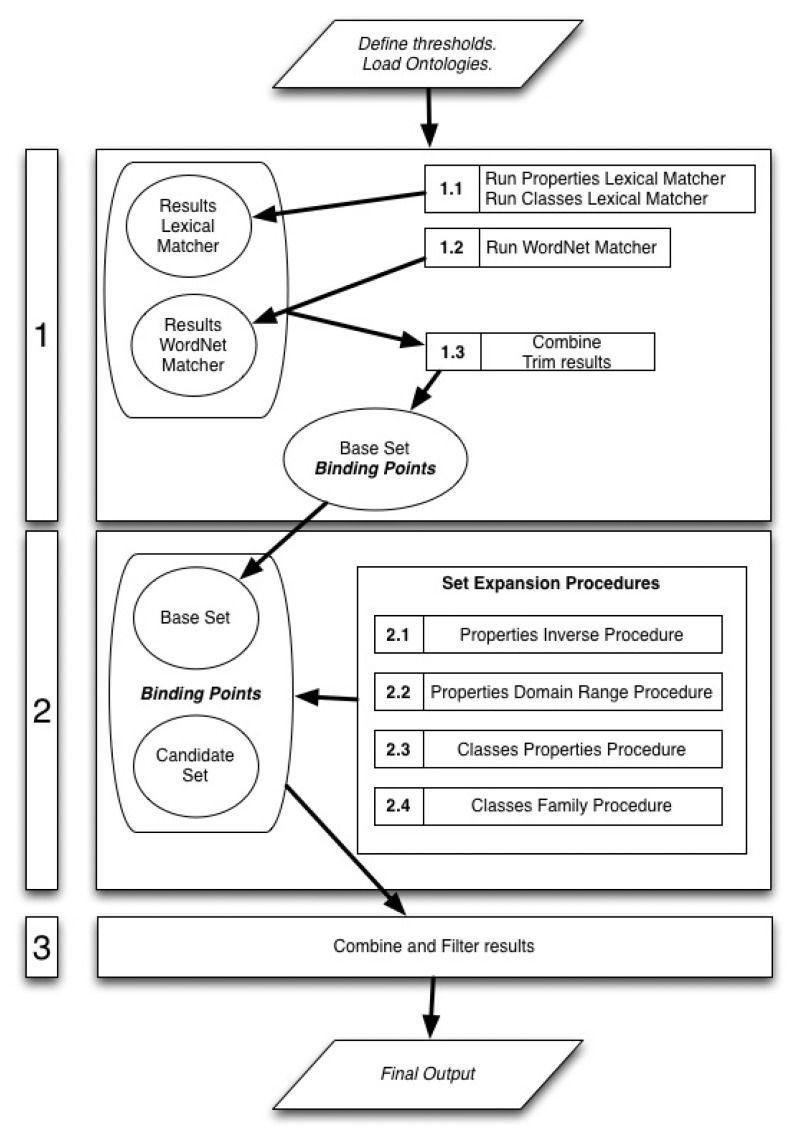
Schematic diagram of algorithm steps.

The method is split into three phases as seen in Figure 2. In the first phase the ontologies are matched using the lexical matchers and the initial *binding points* are discovered. Thereafter, in the second phase the structural matchers are applied to the initial *binding points* aiming at iteratively discovering new ones. In the last phase, the final output of this algorithm is composed by joining and filtering the *binding points* discovered in the previous phases. The structural matchers used in the second phase are defined as independent modules so that the output of a structural matcher is not necessary for the run of any of the others and it does not affect their outputs. The fact that the different structural matchers are independent allows selecting just the most suitable ones for each task.

In the following sections a description of each one of the steps and sub-steps comprising the algorithm is provided. The order of the sections follows the sequence shown in the schematic diagram of the algorithm in Figure 2. For the purpose of this description, we consider that all the structural matchers are used.

### 5.3. Step 1: Obtaining Initial Binding Points

Obtaining the initial *binding points* is a crucial part of the matching process since these are the base on which the rest of the algorithm is built. To identify these initial *binding points* we use lexical matchers, specifically one that uses the WordNet database as external resource and another one that uses string-based distance measures. The idea of using two matchers is that one helps in filtering the results provided by the other and hence to obtain the best possible results. In this case, the amount of results obtained is sacrificed for their quality, since the better these initial *binding points*, the better the quality of the final output.

During this phase, the *lexical matchers* and the *WordNet matcher* are run.

#### 5.3.1. *Sub-Step 1.1:* Lexical Matchers

The lexical aligning process is run in two separate sub-phases where classes and properties are aligned by using the *ClassesLexicalMatcher (CLM)* and the *PropertiesLexicalMatcher (PLM)* respectively.

Both these matching procedures are based on string-similarity comparison methods that detect similar entities between the input ontologies. To do so a new distance measure was introduced.

This new measure was defined according to these considerations:

(1) If the lexical similarity between two strings *s*_1_ and *s*_2_ is to be computed, the first step is to remove the numbers that may appear in the strings. Numbers are omitted since they would only interfere in the final result and would not provide relevant matching information.
(2) After doing so, each string is tokenized by using as delimiters any non-alphabetical character, blank spaces and the uppercasing-lowercasing changes in the word, obtaining this way for each one of the original strings a bag of words, $s_{1}^{\prime }$ and $s_{2}^{\prime }$ respectively.
(3) These bags of words are then compared with the following procedure.
  (a) First, the words shared by the two bags are removed, hence obtaining $s_{1}^{\prime \prime }$ and $s_{2}^{\prime \prime }$.
  (b) If after doing so both bags are empty then the similarity measure with the input strings is 1.0. Otherwise, all the words left in the first bag $s_{1}^{\prime \prime }$ are compared to the words left in the second bag $s_{2}^{\prime \prime }$ by using the ***Jaro-Winkler Distance*** [27] and considering the ***Levenshtein Distance*** [27] as a complementary measure.
  (c) If the Jaro-Winkler distance measure of two words returns a number greater than *α*, and for these words the Levenshtein distance measure returns a number lower or equal than *β*, then the combined similarity value of these words is 1.0. These thresholds, *α* and *β*, have been empirically set to 0.90 and 1.0 respectively.
    (1) (Jaro−Winkler(a,b)>α)∩(Levenshtein(α,β)<β)⇒1.0
  (d) In case Levenshtein distance measure indicates that the shortest word must be completely modified to be matched to the second one, this causes a proportional forfeit in the combined result. Otherwise, the result of the Jaro-Winkler distance measure is stored as result for that pair of words in the bags.
    (2) $$\begin{array}{c} \text{Levenshtein}\left(a,b\right)\geq \text{minLenght}\left(a,b\right)\Rightarrow \\ \text{Jaro}-\text{Winkler}\left(a,b\right)*\left(1-\frac{\text{Levenshtein}\left(a,b\right)}{\text{minLenght}\left(a,b\right)}\right) \end{array}$$
(4) Once every possible pair of words is assigned a value, the final result for the bags is computed. To do so, all partial results are added up considering an improvement factor *ϕ* which is used to strengthen the similarity of the bags that share several words in common.
  (3) $$\begin{array}{r} \text{bagEvaluation}\left(s_{1},s_{2}\right)=\\ \frac{\sum \text{pairEvaluation}\left(a,b\right)+\text{repeatedWords}*\phi }{1+\text{repeatedWords}} \end{array}$$
(5) The same comparison procedure is applied to the original strings, *s*_1_ and *s*_2_. As final result, the best score between the original comparison and the bags comparison, is returned.
  (4) $$\begin{array}{r} \text{LexicalValue}\left(s_{1},s_{2}\right)=\\ \text{max}\left[\text{bagEvaluation}\left(s_{1},s_{2}\right),\text{stringEvaluation}\left(s_{1},s_{2}\right)\right] \end{array}$$

We compute separately the similarity of the different types of entities from the input ontologies, namely, classes, object properties and data properties. By following this procedure we obtain a lexical similarity matrix for each type of entity.

#### 5.3.2. *Sub-Step 1.2:* WordNet Matcher

After running the lexical matchers, the following step is to run the *WordNet Matcher*, as it is reflected in the schematic diagram of algorithm steps in Figure 2. The WordNet matcher outputs a matrix that contains the set of similarity values among each class and property from the source ontology and the target ontology. These similarity values are computed using WordNet's *‘basic Synonymy Similarity’*.

(5) WNM={{WordNetValue(c,c′)}∪{WordNetValue(p,p′)}}

These similarity values are used to asses the accuracy of the values provided by the lexical matchers.

To integrate the WordNet database within the current algorithm the *Java WordNet Library (JWNL)* [84] was used.

#### 5.3.3. *Sub-Step 1.3:* Combine and Select Results

Once the lexical matchers have completed their execution, the resulting matrices are combined in a new structure. In this structure all the candidate correspondences generated by the properties lexical matcher and the classes lexical matcher are joined. As seen in Equation (6) each *Combined Set (CS) tuple* has either a pair of classes or properties from both ontologies together with their corresponding lexical and WordNet values.

(6) CS={(c,c′,CLM(c,c′),WNM(c,c′))}∪{(p,p′,PLM(p,p′),WNM(p,p′))}

Having all these possible initial *binding points* or candidate correspondences provided by the lexical matchers in the same structure eases the sifting out.

The sifting out is determinant for the algorithm since it helps in reducing the number of initial *binding points* that will be used as a starting point for the next step. As we stated before, only the most accurate ones should be used as a base for the next phase of the algorithm, therefore the threshold was set to 1.0, guaranteeing that only the best matches are used.

This sifted set (*Sifted_CS*) represents the base on which the *set expansion procedures* are applied. These expansion procedures aim at discovering new *binding points* between the two input ontologies by exploiting structural features of the ontologies.

### 5.4. Step 2: Discovering New Binding Points

As stated before, the sifted set becomes the *base set* on which the expansion procedures are built. This *base set* contains the initial *binding points* to which new procedures are applied to obtain new candidate *binding points*. These candidate *binding points* can link either properties or classes and therefore there are *properties candidate correspondences* and *classes candidate correspondences*.

Depending on the structural feature that is exploited in each procedure, there is a different likelihood that the discovered candidate *binding points* are promising, therefore some of these procedures will directly update the *base set* by modifying or inserting new *binding points* while others will be kept on a *candidate set*.

Each one of the new discovered *binding points* is assigned a type that identifies the procedure and sub-procedure that led to its discovery. In case one is reached by several procedures all of them are recorded using tags.

These procedures are sequentially applied, and each one of them exploits a different feature of the ontologies.

(1) **Properties Inverse Procedure:** this procedure identifies new property candidate correspondences by exploiting the existence of a defined inverse property in the ontologies, and if so, tags it and adds it to the corresponding set.
(2) **Properties Domain Range Procedure:** this procedure obtains new class candidate correspondences. Within this procedure the domain and range class groups of the properties are assessed. This evaluation is not limited to the first-level domain and range classes, but higher levels are also retrieved and compared, to obtain the most accurate correspondences possible.
(3) **Classes Properties Procedure:** by applying this procedure not only class candidate correspondences but also property candidate correspondences may be discovered. This procedure recursively identifies the similar properties existing among the class candidate correspondences, and then assesses the existence of other classes belonging to the domain or range of this properties that could be a new candidate correspondence.
(4) **Classes Family Procedure:** this last procedure is the familiar approach to identifying new class candidate correspondences. Following this approach class candidate correspondences are retrieved and their familiar relations are exploited. For each pair of classes in the class candidate correspondences, its superclasses, subclasses and sibling classes are recovered, and then assessed to determine if new matches are possible.

These procedures cause the definition of 19 different sets. Each one of them representing a different path to discover correspondences. Each correspondence identifies two classes or properties and the list of all the sets that this correspondence is included in. In case the same correspondence is retrieved in several occasions following the same path, an occurrence value is properly updated. Each branch of each procedure is independently identified and tagged, so that the different correspondences can always be linked to the specific procedures, sub-procedures and branches that retrieve them. This structure of sets is crucial to the performance of the algorithm. Depending on which path is followed to discover a correspondence (which procedure, sub-procedure and branch), its associated accurateness will also be different, as well as the degree of confidence that is applied to them when selecting the correspondences for the final alignment.

### 5.5. Selecting Binding Points

After applying the procedures presented in Section 5.4, a final version of the *base set* and *candidate set* is obtained. In case that any of these methods had caused the modification of the initial *base set* with new *binding points*, a new iteration is done. In this new iteration the *base set* is different from the initial one, because it is composed only by those *binding points* that were inserted in the *base set* in the previous iteration.

Iterations stop when no more modifications are done in the *base set* and the system converges. After finishing all the iterations results are combined and the selecting process begins. This selecting process is essential since it is a way of dismissing false correspondences and therefore defining the final output of the algorithm.

For this selecting process several restrictions have been defined which treat differently class candidate correspondences and property candidate correspondences. These restrictions are based in the idea that the different procedures defined exploit different features of the ontologies and therefore they outcome correspondences with different levels of accuracy. Hence, the tagging of the correspondences allows their selection, in order to choose for the final output the best possible pairs.

The rules implemented at present in the algorithm are far from being the best ones which is why the refinement of the restriction rules is one of the major steps to take in the future evolution of *OntoPhil.*

Some of the basic restriction rules in the algorithm are:

- *Retrieve those correspondences that belong to CC_BASE_SET*. The correspondences in this set have been obtained with the initial binding point calculation.
- *Retrieve those correspondences that belong to CC_DIRECT_DERIVED*. The correspondences in this set have been obtained with the *Properties Domain Range Procedure*, out of those initial relationships that only define a class as domain and as range. If we have a correspondence between two properties obtained from a initial binding point, and each one of these properties only has a domain class and a range class, then these are also correspondences.
- *For those property correspondences that share the same entity in the source ontology, retrieve those whose mean, between the WordNet value and lexical value, is higher*.

After detailing the main steps in our algorithm, in Section 6 we present the experiments used to evaluate it, as well as the results obtained.

## 6. Evaluation

The goal of any algorithm for ontology matching is to generate an alignment that discovers the correspondences, and only the correct ones between two input ontologies. The correctness of these correspondences is evaluated against a reference matching provided by the human interpretation of the meaning of the input ontologies. Although the purpose is clear, in most cases either incorrect correspondences are discovered or correct ones are not.

To evaluate the accuracy of an ontology matching algorithm, the standard information retrieval metrics of *Precision, Recall and F-measure* are used.

The *Precision* measures the ratio of correctly found correspondences over the total number of returned correspondences, which in logical terms reflects the degree of correctness of the algorithm.

(7) $$\text{precision}=\frac{\#\text{true}\_\text{positives}}{\#\text{correspondences}\_\text{found}}$$

The *Recall* measures the ratio of correctly found correspondences over the total number of expected correspondences which in logical terms measures the degree of completeness of the alignment.

(8) $$\text{recall}=\frac{\#\text{true}\_\text{positives}}{\#\text{existing}\_\text{correspondences}}$$

Even though precision and recall are widely used and accepted measures, in some occasions it may be preferable having a single measure to compare different systems or algorithms. Moreover, systems are often not comparable based solely on precision and recall. The one which has a higher recall may have a lower precision and vice versa [17] and therefore the *F-measure* was introduced.

(9) $$f-\text{measure}=\frac{\text{precision}*\text{recall}}{\left(1-\alpha \right)*\text{precision}+\alpha *\text{recall}}$$

We take *α* as 0.5, so the outputted value shows no bias towards *precision* or *recall.*

*OntoPhil* will be integrated in a smart city environment, in which, there is not a mature benchmark available to test the performance of matching algorithms. Hence, we have initially turned to the well-known test sets used at the Ontology Alignment Evaluation Initiative to validate the general performance of our algorithm, detailed in Section 6.1. Next, we have conducted a more specific evaluation focused on validating the performance of *OntoPhil* within the smart city environment, detailed in Section 6.2.

### 6.1. Experiments Using the OAEI Datasets

As usual in the field, we have used the precision, recall and f-measure metrics to assess the accuracy of our algorithm and compared our results with those provided by the algorithms that took part in several tracks of the Ontology Alignment Evaluation Initiative 2013 (OAEI-13), specifically the *benchmark, conference* and *anatomy* tracks.

These tests were run on a Mac OS X machine configured with a 2.6 GHz Intel Core i3 processor and 4GB of RAM.

#### 6.1.1. Benchmark Track

This is a well known series of tests that has been used for several years and which allows for comparison with other systems since 2004. This benchmark is built around a seed ontology and many variations of it [85] and its purpose is to provide a stable and detailed picture of the algorithms. In each one of these tests an ontology is to be matched with another which has suffered certain modifications that affect both the entities and the ontology's structure. As an example of modifications that affect the entities, there is the replacement of the names by random strings, synonyms or expressed using different conventions. Regarding the hierarchy of the entities, the changes include expansion, flattening or complete suppression of entities, instances, *etc*. The purpose of these tests is to evaluate the robustness of the different algorithms.

Table 1 shows the results achieved by *OntoPhil* when tested using the OAEI-13 benchmark test. The systems in this table are ordered according to their highest average *f-measure*. *OntoPhil* behaves in average better than most of the systems competing since the values achieved are good in the three measures and it improves the results of the rest of algorithms in at least one of them. It is worth noticing that several systems did not complete all the tests, even if they were able to do so in the OAEI-12 campaign.

In this table, apart from the results of the competing algorithms, a baseline matcher *edna* was included as reference. Regarding this division created by edna, *OntoPhil* is placed among the best performing algorithms, only outperformed, in terms of *f-measure*, by *IAMA* and *YAM*++.

#### 6.1.2. Conference Track

The conference track presents the problem of finding alignments within 16 ontologies describing the domain of conference organization. These ontologies have been based on actual conference series and their web pages, actual software tools for conference organization and the experience of people dealing with conference organization [86]. Due to the heterogeneous origin of the ontologies, it is more difficult to produce correct alignments between the input ontologies than it is with the ontologies in the benchmark track.

Table 2 gathers the results achieved when using the OAEI-13 conference track, also ordered according to the highest average *f-measure*. The results in this table were obtained using the *original reference alignment (ra1)* as gold standard, as there is also an *entailed reference alignment (ra2)* generated as a transitive closure from the original reference alignment, which is not available [87]. For those systems that were run using different configurations, the average values were computed to be shown in Table 2.

In this table two baseline matchers, *edna* and *StringEquiv*, divide the systems competing into three groups. Those performing worse than StringEquiv, those better than StringEquiv but worse than edna and finally, those outperforming both. *OntoPhil* is situated in the latter of these groups.

#### 6.1.3. Anatomy Track

The anatomy track consists of aligning the Adult Mouse Anatomy [88] and the NCI Thesaurus [89] which describes the human anatomy. These ontologies are characterized by their size (2744 and 3304 classes respectively), the use of technical terms and the limited use of natural language. This track aims to reflect the real world use case of aligning large ontologies from the biomedical domain. This track is the largest dataset in the OAEI.

Table 3 presents the results achieved using the OAEI-13 anatomy track, ordered according to the highest average *f-measure*. For those systems that were run using different configurations, the average values were computed to be shown in this table. This is the case of *AML* and *GOMMA* which have both two versions that use background knowledge, specifically they reuse previous mappings between UMLS [90], Uberon [91] and FMA [92].

The systems that took part this track were given a time deadline to provide the results [93]. To comply with these requirements, certain timeouts were added to the algorithm, and since, the results obtained do not reach the quality of those obtained in benchmark and conference tracks.

### 6.2. Experiments with SOFIA

As the development of the platform is not completed yet, it was impossible to test this algorithm with a mature test set coming from the environment where it will be deployed or to compare it with other algorithms defined for the same purpose. To asses the validity and usefulness of *OntoPhil* in the environment where it will be deployed, *i.e.*, a smart city described using the *SOFIA* ontology, we have defined and conducted some experimental tests. Each one of these tests involves matching the *SOFIA* ontology to an agent ontology.

#### 6.2.1. Research Questions

With these experiments we aim to determine if it is possible to automatically match the ontologies of the different types of agents to the smart city's ontology, and if the results obtained from the matching are useful in terms of performance and compliance.

- Is it possible to use ontology matching to avoid human involvement when new agents are included in a smart city?
- Is it possible to seamlessly exchange the essential operational information between the agents and the smart city?

#### 6.2.2. Experiments’ Design

The experiments defined involve matching the agents’ ontologies described in Table 4 to the *SOFIA* smart city's ontology. This ontology defines 197 classes, such as *Activity, AlertEvent*, *Device, EvacuationOrder, EventManager, Observation, Role* or *Sensor* and 246 properties, such as *hasConnectionProtocol*, *hasMeasurement*, *hasParameter*, *isAssociatedToEvent*, *playsRole* or *subscribesFor*. The objective of such ontology is to improve a city's monitoring and control capabilities by exploiting the broad range of information on city conditions that can be obtained by integrating motley monitoring systems.

Some of the agents’ ontologies used for these tests are part of different deployments of pilot subsystems within *SOFIA*^2^, the remaining were obtained online. To retrieve these agents’ ontologies we have looked into websites that detail current and past initiatives, projects and deployments where ontologies are used to describe their knowledge. These agents’ ontologies were sorted to dismiss those that showed no relevance in a smart city environment. These agents’ ontologies were adapted to OWL format, whenever necessary, to be used as input in *OntoPhil*. Apart from this modification, the agents’ ontologies remain as defined by their developers.

Finally, we had 39 different ontologies to run the tests. These ontologies represent different agents that need to interact with the smart city. These ontologies cover different types of sensor agents, devices and systems. Table 4 summarizes the information about ontologies’ sizes. This table identifies the name of each ontology, its type and the amount of classes and properties it has.

These ontologies can be divided into the following categories:

- *Devices*: These ontologies represent different devices that could be plugged into a smart city, such as *deviceSSDS*, that represents a marine device or *IoT.est-Resource*, that represent a resource in an Internet of Things environment or *RobotOntology* that describes a robot programming ontology.
- *Sensors*: These ontologies represent agents that include sensors to measure different parameters, such as temperature, noise, rain, *CO*_2_, ambient humidity, luminosity, etc.
- *Systems*: These ontologies represent systems that could be connected to the smart city, such as and *SEFF*, that is a streamflow measuring system or *oboe-beta* and *oboe-sbclter*, that belong to an online resource for managing ecological data and information. This category also includes the ontologies representing agents that provide a service, as it is the case of *IoT.est-Service*, which represents a general service in an Internet of Things environment.

For each one of these ontologies, two different reference alignments were defined by a team of researchers within our group, the *reference alignment* and the *essential reference alignment*, being the latter a subset of the first.

The *reference alignment* allows the computation of the compliance measures, and helps in determining whether the algorithm can be used in this environment or not. On the other hand, the *essential reference alignment* only includes those mappings that are crucial to allowing the agent to transmit, its sensed information or its request, to the smart city.

By defining two reference alignments we aim at determining whether the automatic exchange of the essential information between the smart city and the agents is feasible or not, because achieving high compliance measures is not relevant if the algorithm fails to determine the core information that must be shared.

In the essential reference alignment, the mappings included are just those devoted to identifying (i) the type of agent; (ii) the result, value, observation or event that it has to transmit; (iii) its location; and (iv) its constraints (threshold, unit or range).

**Figure 3.**
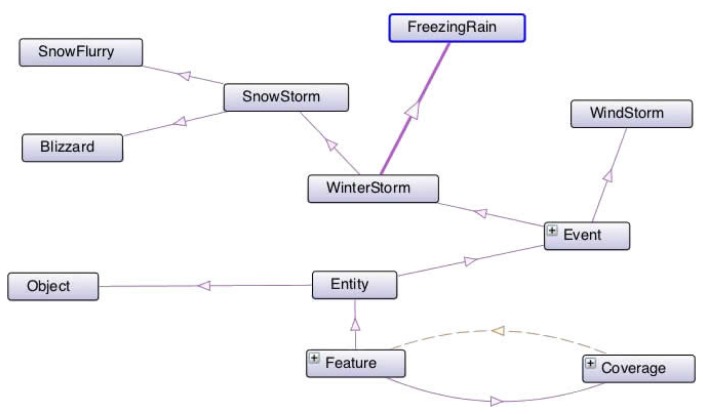
Snippet of *sensor-observation* agent.

For example, let us consider the ontology *sensor-observation*. Its reference alignment, presented in Table 5, includes more correspondences than its essential reference alignment, presented in Table 6, because for exchanging the measurements and results from the sensors represented in this ontology, the remaining correspondences are not necessary. A snippet of this ontology is shown in Figure 3.

#### 6.2.3. Results

These tests were run on a Mac OS X machine configured with a 2.6 GHz Intel Core i3 processor and 4GB of RAM. Table 7 shows the general information about the ontologies, together with the average runtime results of 5 runs and the standard deviation. We report time measurements in seconds.

As we have previously stated, we compute two sets of *precision, recall* and *f-measure* values, using the *reference alignment* and the *essential reference alignment* respectively.

In Table 8, we show the composition of the reference alignment for each agent's ontology, indicating its number of classes and properties correspondences. We also contrast these values with those obtained by *OntoPhil*, specifying as well the amount of classes and properties correspondences and, the number of missing and extra correspondences retrieved. This table is sorted by average *f-measure*.

The results obtained with these set of agents’ ontologies are very promising as in 82.06% of the cases the *f-measure* value was higher than 0.75. The lowest value of *precision* (0.44) was achieved with the *personasonto* ontology which incorporates concepts and properties used to model personas (a person's perceived or evident personality, as that of a well-known official, actor, or celebrity; personal image or public role) and which is not directly relevant to the smart city environment. On the other hand, the lowest *recall* value, corresponds to the *SEFF* ontology that was designed to model provenance in a workflow for streamflow forecasting.

If we further analyze these results, *OntoPhil* missed to retrieved only 20.5% of the correspondences, which would need to be manually added. The amount of those that would need to be manually dismissed is even lower, 11.9%. This figures support the hypothesis that, in most situations, it is possible to avoid human input when including a new agent in the smart city.

The results obtained using the essential reference alignment are summarized in Table 9, in which, we have detailed the number of classes and properties correspondences for both, the essential reference alignment and the *OntoPhil* output. Additionally, just for *OntoPhil* we have included the amount of missing and extra correspondences detected. Finally, as in Table 8, we have computed the compliance measures of *precision, recall* and *f-measure*.

These results show that in 71.8% of the cases the highest value (1.0) is achieved. The lowest *precision* and *recall* values are obtained, with the *personasonto* and *SEFF* ontologies, as happened using the reference alignment (see Table 8). The human input in this case is even lower than using the reference alignment, as the amount of missing correspondences falls to 6.13%, and the amount of missing ones to 4.9%. Additionally, it is worth noticing that, these modifications are concentrated in the same ontologies. These figures support the premise that it is possible to exchange the essential operational information between the agents and the smart city.

In spite of the promising results obtained from these experiments, the validation of this algorithm in a real environment remains as the most important task in the near future, as it will allow us to further refine and improve the algorithm.

In this section we have described the experiments used to test *OntoPhil* as well as the results obtained. In the next section, we further review and discuss the main contributions of this paper.

## 7. Discussion

In this section we address the main points of the proposal presented in this work. Such proposal encompasses the utilization of ontology matching techniques within the smart city environment and the description of a novel matching algorithm.

As this proposal is part of an ongoing development there are still issues to be addressed which are the main goals of the present and future efforts. The computation of the initial set of binding points relies completely on the results of our lexical matcher, therefore if the entities to be matched share no lexical relation, the algorithm will not output satisfactory results.

Additionally it is worth reflecting on the fact that the reference alignments used to calculate the precision, recall and f-measure values are usually user-defined, and we have found that our algorithm outputs candidate alignments that are semantically sound and yet not considered in the reference alignment. Although the number of such results is small regarding the total outputted alignments, the fact that these actually exist is by itself a situation worth studying.

As we have already introduced in Section 4, our algorithm shares certain similarities with others that also start their alignment discovery by computing some initial binding points, or as known in these works, anchors. All these systems take these anchors and exploit them to output the final alignment. In our algorithm this general outline is also followed although there are fundamental differences in the way we compute the initial alignments and also in the way we exploit them.

In *Anchor-Flood*, for instance, the module that obtains the initial anchors is not considered as a part of the algorithm. This means that this approach does not start with two input ontologies but it does already with some externally provided anchors. In terms of complexity, scalability, runtime computation, *etc.*, this feature makes the algorithms fundamentally different. Besides obtaining these initial anchors both lexical and statistical relational information is used. Conversely, in our algorithm, the process of obtaining the initial binding points is a part of the algorithm which takes two input ontologies and by applying terminological methods it obtains a set of initial pairs to be expanded.

Regarding the *Anchor-Prompt* approach, there are several differences worth noticing. First of all there is the fact that the initial anchors can be user-provided or automatically obtained with a lexical matcher. In case this algorithm is fed with user-defined pairs the results will not be meaningful from a comparative point of view, as human beings can identify as pairs entities that would be hard to obtain in an automatically defined way. Additionally, out of these initial anchors, this algorithm constructs some paths between anchors and the similarity measure of the pairs of classes it encounters in the paths comes from the aggregation of the similarity values from all the paths these show up (cumulative score). It returns as a match those pairs that surpass a certain threshold value. Finally, another fundamental difference is that this approach only discovers correspondences for class entities, not for relations. In our algorithm the way the initial pairs are exploited is quite different, starting with the fact that we also consider as initial binding points pairs of relations. Our binding points are seen as connection points between both ontologies and so the neighborhood of those points is analyzed in terms of classes and relations, but in an independent way from the rest of binding points. Additionally our algorithm was designed to align a large ontology with some smaller ones.

Another related approach mentioned before is *ASCO* whose main difference with *OntoPhil* relies in the fact that both measures that *ASCO* computes, lexical and structural, are combined in a single value, and only those pairs above a certain threshold are selected for the final alignment. In our approach, the lexical matcher is used to identify the initial binding points which are then structurally exploited by means of different procedures. The new binding points discovered by these procedures are tagged, and it is only in the final step when we filter all the candidate pairs relying on the tags that these have and which reflect the procedures that led to their discovery, considering that some procedures are more likely to produce valid results than others. We do not combine the lexical and structural values as there is actually not a structural value related to the pairs but a list of tags. Another difference is found in the way *ASCO* separately computes correspondences for classes and for properties.

In the case of *Eff2Match*, in its candidate generation step, the algorithm identifies candidates for those entities in the source ontology that have not been matched in the initial anchor generation step. Despite the fact that the initial anchors or binding points are lexically obtained in both algorithms, we also consider these as candidates and rely on them to discover new ones. Additionally in *Eff2Match* the anchor expansion is based on terminologically comparing the entities in the source ontology with their candidate entities. Finally, this algorithm requires of user interaction as there is a set of parameters that must be manually tuned, which, as described by the authors, may be a tedious and ineffective procedure.

*LogMap* has an outline that completely fits in with ours, and here the difference is to be found in the individual steps within the general outline, as their procedures are more complex than those we propose. Indeed, out of all the systems that share the same set of principles this is the most complex one. To mention some differences, the procedure to compute the anchors is different as LogMap initially does a lexical and structural indexation. Once these indexes are complete the initial anchors are obtained by intersecting the lexical indexes of each input ontology. The similarity value of the anchors is based on the locality principle, so if the neighbors of the pair of classes in an anchor have a low confidence value, that anchor may be dismissed. In our approach, the initially obtained binding points are fixed and can not be dismissed unlike the others that are discovered by the different procedures.

Finally, the *SOBOM* approach is based on the extraction of sub-ontologies out of the initial anchors. One major difference is found in the information exploited to obtain these anchors, since *SOBOM* uses in addition to labels, names, and ids, other aspects such as the number of constraints, individuals, *etc.* Another distinguishing aspect is found in how similarity is calculated. In *SOBOM* the concept similarity is obtained from the sub-ontologies derived from both input ontologies according to their depths. These results are later used to obtain the relationship alignments. In *OntoPhil* there is not this sequencing, indeed, the correspondences between properties are treated as any other binding point.

As these approaches we have just discussed, *OntoPhil* is another one whose main novelty relies on the field where it is applied, smart cities. This is a relatively new field of work and therefore the number of solutions addressing it is still low. It is highly likely that if the smart city field continues to evolve as heretofore, new approaches will arise, some that may follow the lead that we are describing in this work but also others that may propose completely new alternatives.

Other algorithms with the same purpose may be defined in the future or existing ones can be revisited and adapted. With *OntoPhil* we have tried to develop an algorithm adapted to the environment where it will be deployed as the quality of the final alignment, even if important it is not as much as obtaining results in real time with a lightweight algorithm that can be implemented in the agents.

This algorithm has been tested using the *OAEI datasets* and the *SOFIA ontology*, however, as the deployment of this platform will not be completed until mid-2015, the evaluation in the final scenario is not possible yet and which remains as one of the main steps to take.

Focussing on the results obtained using the agents’ ontologies and the smart city's ontology, when describing these experiments we have aimed at testing the different types of agents that may interact in the smart city, however the number of these tests is not balanced as it was easier to find ontologies devoted to describing potential agents than to describing potential subsystems. The results obtained in these tests are complemented by those obtained using the OAEI, which encourage the further development of this proposal.

This discussion section we discuss our main findings. Next, in the final section, we outline the most important conclusions obtained from this work, and point out the future lines of research.

## 8. Conclusions and Future Work

In this paper we have described our proposal of using ontology matching techniques within the smart city field. For this purpose, we have developed a novel ontology matching algorithm. The use of ontology matching techniques in smart cities is a novel approach, however it fits to the nature of problem to tackle and the parties involved. In a smart city the amount of information retrieved is remarkably big as well as the number and variety of devices that gather it. In spite of this, for a smart city to be functional and useful for its users it must be open and seamlessly provide services to its users, regardless of the devices users choose to interact with it. To comply with this requirements, those approaches that force the use of a certain knowledge representation or those that rely on human input are not suitable. This is the main reason that grounds our approach.

Using *OntoPhil*, the knowledge representations of the different parties do not need to be modified, as the algorithm detects the semantical equivalences between the entities, which are used for the exchange of information between agents and the smart city. Besides, this algorithm was designed to avoid any kind of human input, since it discovers correspondences among entities of input ontologies by exploiting both their lexical and structural information.

*OntoPhil* was defined to allow the interaction of the agents in a smart city in a transparent way and regardless of the system for which each agent was designed. By taking this ontology matching based approach to information fusion we secure the interoperability of the parties in the multi-agent system and lay the foundations for an automated integration of new agents.

This algorithm has been tested using the version for 2013 of the benchmark, conference and anatomy test suites of the OAEI campaign. We have contrasted our results with those provided by the systems that took part in that contest, and as tables in Section 6 reflect the results are quite good. We have also designed and conducted an experiment to test the performance of *OntoPhil* when using ontologies from the smart city domain. All this encourages the capability of *OntoPhil* in the field of AmI for integrating knowledge from different sources.

As we outlined in Section 7, in spite of the good results of this line work, there are still several steps that must be taken before it is fully functional. First, there is work to do to improve the algorithm. Some tasks are, for instance, the calculation of the initial binding points, the refinement of the restriction rules so the amount of false positives retrieved in the alignment are kept to a minimum or the inclusion other features such as multilingual support and instance matching. These improvements will be developed to be included in the algorithm as a way to enhance the results of *OntoPhil*.

There is also the need to validate the approach by testing it within the *SOFIA*^2^ final deployment. These real-environment tests will be very useful to test the robustness, scalability and flexibility of the algorithm before the final deployment.

## Figures and Tables

**Table 1.** Results obtained in the OAEI-13 benchmark track compared with OntoPhil (ordered by F-measure).

	*MaPSSS*	*StringsAuto*	*RiMOM2013*	*ServOMap*	*LogMapLt*
P	0.84	0.84	0.49	0.53	0.43
F	0.14	0.14	0.19	0.33	0.46
R	0.08	0.08	0.12	0.22	0.50
	*MaasMatch*	*OntoK*	*LogMap*	*XMapGen*	*edna*

P	0.66	0.69	0.72	0.66	0.58
F	0.50	0.51	0.51	0.52	0.54
R	0.41	0.40	0.39	0.44	0.50
	*WeSeE*	*AML*	*XMapSig*	*Synthesis*	*CIDER-CL*

P	0.96	1.00	0.71	0.60	0.84
F	0.55	0.57	0.59	0.60	0.66
R	0.39	0.40	0.50	0.60	0.55
	*CroMatcher*	*Hertuda*	*Hotmatch*	*WikiMatch*	*ODGOMS*

P	0.75	0.90	0.96	0.99	0.98
F	0.68	0.68	0.68	0.69	0.70
R	0.63	0.54	0.50	0.53	0.54
	**OntoPhil**	*IAMA*	*YAM*++		

P	1.00	0.99	0.84		
F	0.71	0.73	0.77		
R	0.62	0.57	0.70		

**Table 2.** Results obtained in the OAEI-13 conference track compared with OntoPhil (ordered by F-measure).

	*MaasMatch*	*CroMatcher*	*RIMOM2013*	*XMapGen*	*StringEquiv*
P	0.28	0.52	0.59	0.63	0.80
F	0.37	0.51	0.54	0.54	0.56
R	0.55	0.50	0.49	0.50	0.43
	*XMapSig*	*SYNTHESIS*	*CIDER_CL*	*OntoK*	*HotMatch*

P	0.76	0.77	0.75	0.77	0.71
F	0.56	0.57	0.58	0.58	0.59
R	0.44	0.45	0.47	0.47	0.51
	*IAMA*	*LogMapLite*	*WikiMatch*	*edna*	*HerTUDA*

P	0.78	0.73	0.73	0.76	0.74
F	0.59	0.59	0.59	0.60	0.60
R	0.48	0.50	0.49	0.49	0.50
	*WeSeE-Match*	*MapSSS*	*ServOMap*	*ODGOMS*	*StringsAuto*

P	0.85	0.83	0.73	0.75	0.78
F	0.61	0.62	0.63	0.64	0.64
R	0.47	0.50	0.55	0.56	0.54
	**OntoPhil**	*LogMap*	*AML*	*YAM*++	

P	0.86	0.80	0.87	0.80	
F	0.67	0.68	0.69	0.74	
R	0.57	0.59	0.57	0.69	

**Table 3.** Results obtained in the OAEI-13 anatomy track compared with OntoPhil (ordered by F-measure).

	*MaasMatch*	*WeSeE*	*HerTUDA*	*CIDER-CL*	*IAMA*
P	0.36	0.62	0.69	0.65	1.00
F	0.41	0.47	0.68	0.69	0.71
R	0.48	0.38	0.67	0.73	0.56
	*XMapGen*	*ServOMap*	*XMapSig*	*StringEquiv*	**OntoPhil**

P	0.81	0.96	0.86	1.00	1.00
F	0.75	0.75	0.75	0.77	0.77
R	0.70	0.62	0.67	0.62	0.63
	*HotMatch*	*WikiMatch*	*ODGOMS*	*MapSSS*	*LogMapLite*

P	0.98	0.99	0.98	0.90	0.96
F	0.77	0.80	0.82	0.83	0.83
R	0.64	0.67	0.71	0.77	0.73
	*StringsAuto*	*LogMap*	*GOMMA*	*YAM*++	*AML*

P	0.90	0.92	0.94	0.94	0.95
F	0.84	0.88	0.90	0.91	0.91
R	0.78	0.85	0.86	0.87	0.88

**Table 4.** General information about the agents’ ontologies used for the experiments with *SOFIA*.

***Name***	***Type***	***Classes***	***Properties***	***Total***
*context-mobile*	Device	15	39	54
*device*	Device	53	2	55
*deviceSSDS*	Device	4	3	7
*IoT.est-Resource*	Device	8	15	23
*personasonto*	Device	52	66	118
*RobotOntology*	Device	95	40	135

*cesn*	Sensor	46	20	66
*context*	Sensor	164	97	261
*gasSensor*	Sensor	9	7	16
*gasSensor*-2	Sensor	13	9	22
*generalAgent*	Sensor	24	30	54
*ground-context*	Sensor	11	18	29
*humiditySensor*	Sensor	6	4	10
*humiditySensor*-2	Sensor	18	9	27
*luminositySensor*	Sensor	6	6	12
*luminositySensor*-2	Sensor	11	13	24
*noiseSensor*	Sensor	5	5	10
*noiseSensor*-2	Sensor	10	7	17
*oceansevents*	Sensor	47	23	70
*presenceSensor*	Sensor	5	3	8
*presenceSensor*-2	Sensor	12	5	17
*pressureSensor*	Sensor	2	3	5
*pressureSensor*-2	Sensor	12	12	24
*rainSensor*	Sensor	4	4	8
*rainSensor*-2	Sensor	11	8	19
*Sensor*	Sensor	16	10	26
*sensor-observation*	Sensor	42	45	87
*smokesensortest*	Sensor	4	4	8
*smokesensortest*-2	Sensor	6	5	11
*tempsensor*	Sensor	1	2	3
*tempsensor*-2	Sensor	10	3	13
*windSensor*	Sensor	3	4	7
*windSensor*-2	Sensor	6	7	13

*IliumReport*	System	38	58	96
*IoT.est-Service*	System	24	55	79
*oboe-beta*	System	29	31	60
*oboe-sbclter*	System	496	1	497
*SEFF*	System	11	15	26
*ssnExtended*	System	122	149	271

**Table 5.** Reference alignment for *sensor-observation*.

***Sensor-Observation***	***Smartcity***
Event	Event
Observation	Observation
PropertyType	AbstractCharacteristic
ResultData	MeasurementResult
System	System
memberOf	isMemberOf
observedProperty	observedCharacteristic
parameter	hasParameter
result	hasMeasurement

**Table 6.** Essential reference alignment for *sensor-observation*.

***Sensor-Observation***	***Smartcity***
Observation	Observation
ResultData	MeasurementResult
observedProperty	observedCharacteristic
result	hasMeasurement

**Table 7.** Runtime results.

***Name***	***Classes***	***Properties***	***Total***	***Runtime(s)***	***SD***
*pressureSensor*	2	3	5	0.92	0.01
*humiditySensor*	6	4	10	2.03	0.01
*tempsensor*	1	2	3	2.66	0.08
*noiseSensor*	5	5	10	3.68	0.03
*windSensor*	3	4	7	3.72	0.02
*presenceSensor*	5	3	8	3.76	0.03
*deviceSSDS*	4	3	7	3.87	0.07
*humiditySensor-2*	18	9	27	4.02	0.05
*oceansevents*	47	23	70	4.24	0.53
*luminositySensor*	6	6	12	4.59	0.06
*gasSensor*	9	7	16	4.84	0.04
*tempsensor-2*	10	3	13	4.85	0.03
*luminositySensor-2*	11	13	24	4.91	0.04
*rainSensor*	4	4	8	5.54	0.06
*windSensor-2*	6	7	13	5.56	0.04
*gasSensor-2*	13	9	22	5.76	0.05
*smokesensortest-2*	6	5	11	6.47	0.05
*noiseSensor-2*	10	7	17	6.52	0.06
*smokesensortest*	4	4	8	6.80	0.08
*Sensor*	16	10	26	7.30	0.06
*rainSensor-2*	11	8	19	7.51	0.07
*presenceSensor-2*	12	5	17	7.74	0.08
*pressureSensor-2*	12	12	24	10.16	0.09
*generalAgent*	24	30	54	13.77	0.22
*IoT.est-Resource*	8	15	23	13.80	0.16
*ground-context*	11	18	29	14.50	0.22
*SEFF*	11	15	26	14.70	0.27
*context*	164	97	261	17.67	0.20
*context-mobile*	15	39	54	31.38	1.52
*oboe-beta*	29	31	60	37.22	2.72
*ssnExtended*	122	149	271	49.91	0.61
*cesn*	46	20	66	60.21	0.81
*device*	53	2	55	63.31	1.84
*IoT.est-Service*	24	55	79	65.15	3.56
*RobotOntology*	95	40	135	65.61	1.09
*personasonto*	52	66	118	68.04	0.58
*sensor-observation*	42	45	87	72.06	0.61
*IliumReport*	38	58	96	74.32	0.95
*oboe-sbclter*	496	1	497	409.11	4.88

**Table 8.** Results obtained using the reference alignment.

	**Reference Alignment**			**OntoPhil Output**					**Measures**		

**Name**	**Classes Corresp.**	**Properties Corresp.**	**Total Corresp.**	**Classes Corresp.**	**Properties Corresp.**	**Missing Corresp.**	**Extra Corresp.**	**Total Corresp.**	**Precision**	**Recall**	**F-measure**
*SEFF*	3	5	8	1	1	6	0	2	1.00	0.25	0.40
*context-mobile*	1	2	3	0	1	2	0	1	1.00	0.33	0.50
*IoT.est-Resource*	2	5	7	2	1	4	1	4	0.75	0.43	0.55
*context*	3	4	7	1	2	4	0	3	1.00	0.43	0.60
*sensor-observation*	5	4	9	4	3	2	7	14	0.50	0.78	0.61
*personasonto*	2	2	4	2	2	0	5	9	0.44	1.00	0.62
*ground-context*	4	5	9	2	3	4	0	5	1.00	0.56	0.71
*gasSensor*	6	3	9	3	3	3	1	7	0.86	0.67	0.75
*RobotOntology*	8	5	13	7	4	2	4	15	0.73	0.85	0.79
*device*	7	2	9	5	1	3	0	6	1.00	0.67	0.80
*oceansevents*	1	1	2	1	1	0	1	3	0.67	1.00	0.80
*humiditySensor-2*	9	4	13	7	3	3	1	11	0.91	0.77	0.83
*noiseSensor-2*	6	4	10	5	3	2	1	9	0.89	0.80	0.84
*deviceSSDS*	3	1	4	3	0	1	0	3	1.00	0.75	0.86
*rainSensor*	2	2	4	2	1	1	0	3	1.00	0.75	0.86
*noiseSensor*	5	3	8	3	3	2	0	6	1.00	0.75	0.86
*cesn*	4	5	9	4	5	0	3	12	0.75	1.00	0.86
*IliumReport*	1	2	3	2	1	1	1	4	0.75	1.00	0.86
*pressureSensor*	2	2	4	2	2	0	1	5	0.80	1.00	0.89
*windSensor-2*	5	5	10	5	3	2	0	8	1.00	0.80	0.89
*generalAgent*	13	6	19	11	6	2	2	19	0.89	0.89	0.89
*smokesensortest-2*	6	3	9	6	2	1	1	9	0.89	0.89	0.89
*presenceSensor-2*	7	3	10	7	2	1	1	10	0.90	0.90	0.90
*windSensor*	3	3	6	3	2	1	0	5	1.00	0.83	0.91
*presenceSensor*	3	2	5	3	2	0	1	6	0.83	1.00	0.91
*Sensor*	3	3	6	2	3	1	0	5	1.00	0.83	0.91
*gasSensor-2*	9	4	13	8	4	1	1	13	0.92	0.92	0.92
*ssnExtended*	8	6	14	8	4	2	0	12	1.00	0.86	0.92
*pressureSensor-2*	5	4	9	4	4	1	0	8	1.00	0.89	0.94
*rainSensor-2*	6	4	10	6	4	0	1	11	0.91	1.00	0.95
*luminositySensor-2*	6	5	11	6	4	1	0	10	1.00	0.91	0.95
*oboe-beta*	4	6	10	4	6	0	1	11	0.91	1.00	0.95
*tempsensor*	1	2	3	1	2	0	0	3	1.00	1.00	1.00
*Smokesensortest*	3	2	5	3	2	0	0	5	1.00	1.00	1.00
*humiditySensor*	4	2	6	4	2	0	0	6	1.00	1.00	1.00
*luminositySensor*	3	4	7	3	4	0	0	7	1.00	1.00	1.00
*tempsensor-2*	7	2	9	7	2	0	0	9	1.00	1.00	1.00
*IoT.est-Service*	2	2	4	2	2	0	0	4	1.00	1.00	1.00
*oboe-sbclter*	1	1	2	1	1	0	0	2	1.00	1.00	1.00

**Table 9.** Results obtained using the essential reference alignment.

	**Essential Alignment**			**OntoPhil Essential Output**					**Measures**		

***Name***	**Classes Corresp.**	**Properties Corresp.**	**Total Corresp.**	**Classes Corresp.**	**Properties Corresp.**	**Missing Corresp.**	**Extra Corresp.**	**Total Corresp.**	**Precision**	**Recall**	**F-measure**
*personasonto*	1	0	1	1	0	0	2	3	0.33	1.00	0.50
*SEFF*	1	4	5	1	1	3	0	2	1.00	0.40	0.57
*context-mobile*	1	1	2	0	1	1	0	1	1.00	0.50	0.67
*IoT.est-Resource*	1	0	1	1	0	0	1	2	0.50	1.00	0.67
*context*	2	2	4	1	1	2	0	2	1.00	0.50	0.67
*device*	2	2	4	1	1	2	0	2	1.00	0.50	0.67
*IliumReport*	1	0	1	1	0	0	1	2	0.50	1.00	0.67
*sensor-observation*	2	2	4	2	2	0	2	6	0.67	1.00	0.80
*rainSensor*	2	2	4	2	1	1	0	3	1.00	0.75	0.86
*cesn*	2	2	4	2	2	0	1	5	0.80	1.00	0.89
*RobotOntology*	4	2	6	4	2	0	1	7	0.86	1.00	0.92
*ground-context*	1	1	2	1	1	0	0	2	1.00	1.00	1.00
*gasSensor*	2	1	3	2	1	0	0	3	1.00	1.00	1.00
*oceansevents*	1	1	2	1	1	0	0	2	1.00	1.00	1.00
*humiditySensor-2*	3	3	6	3	3	0	0	6	1.00	1.00	1.00
*noiseSensor-2*	3	3	6	3	3	0	0	6	1.00	1.00	1.00
*deviceSSDS*	1	0	1	1	0	0	0	1	1.00	1.00	1.00
*noiseSensor*	3	3	6	3	3	0	0	6	1.00	1.00	1.00
*pressureSensor*	2	2	4	2	2	0	0	4	1.00	1.00	1.00
*windSensor-2*	3	2	5	3	2	0	0	5	1.00	1.00	1.00
*generalAgent*	5	4	9	5	4	0	0	9	1.00	1.00	1.00
*smokesensortest-2*	2	2	4	2	2	0	0	4	1.00	1.00	1.00
*presenceSensor-2*	3	2	5	3	2	0	0	5	1.00	1.00	1.00
*windSensor*	3	3	6	3	3	0	0	6	1.00	1.00	1.00
*presenceSensor*	3	2	5	3	2	0	0	5	1.00	1.00	1.00
*Sensor*	2	1	3	2	1	0	0	3	1.00	1.00	1.00
*gasSensor-2*	2	3	5	2	3	0	0	5	1.00	1.00	1.00
*ssnExtended*	4	3	7	4	3	0	0	7	1.00	1.00	1.00
*pressureSensor-2*	2	3	5	2	3	0	0	5	1.00	1.00	1.00
*rainSensor-2*	3	2	5	3	2	0	0	5	1.00	1.00	1.00
*luminositySensor-2*	3	3	6	3	3	0	0	6	1.00	1.00	1.00
*oboe-beta*	2	2	4	2	2	0	0	4	1.00	1.00	1.00
*tempsensor*	1	2	3	1	2	0	0	3	1.00	1.00	1.00
*smokesensortest*	3	1	4	3	1	0	0	4	1.00	1.00	1.00
*humiditySensor*	3	2	5	3	2	0	0	5	1.00	1.00	1.00
*luminositySensor*	3	4	7	3	4	0	0	7	1.00	1.00	1.00
*tempsensor-2*	3	2	5	3	2	0	0	5	1.00	1.00	1.00
*IoT.est-Service*	1	1	2	1	1	0	0	2	1.00	1.00	1.00
*oboe-sbclter*	1	0	1	1	0	0	0	1	1.00	1.00	1.00
